# Cases Illustrating the Surgery of the Thyroid Body

**Published:** 1888-12

**Authors:** W. H. Harsant

**Affiliations:** Surgeon to the Bristol Royal Infirmary


### Clinical Records.

---

CASES ILLUSTRATING THE SURGERY OF THE THYROID BODY. By W. H. HARSANT, F.R.C.S., Surgeon to the Bristol Royal Infirmary.

Enlargements of the thyroid body may be classified as follows:—

1st. Vascular.
2nd. Cystic.
3rd. Adenoid.
4th. Fibrous.
5th. Malignant.

In the first variety, such as we meet with in Graves's disease, local treatment seems of little avail, but in all the other kinds surgical treatment of one kind or other may be of value, and it is a problem of great interest to decide under what conditions each form of treatment should be adopted. Upon the treatment of the cystic variety much has been lately said. Some surgeons, such as Sir Morell Mackenzie and Mr. Mark Hovell, recommend injection of the cyst with a solution of perchloride of iron until suppuration has been produced, and then treating the abscess thus formed by free drainage; and others, as Mr. Mayo Robson, advise an incision into the sac and then suturing the cyst wall to the edges of the skin.*

I have never been called upon to treat a case of this

* My colleague, Mr. Paul Bush, has lately treated a case of large cystic goitre in a man by this means, but it is too early yet to tell the result.

variety, so I do not propose to discuss it now; but in the treatment of the third variety, or simple hypertrophy, we have a choice of several measures:

1st. We may content ourselves with local applications, such as iodine in some form, or the biniodide of mercury combined with the direct rays of the sun! as advocated by Dr. Monat, and stated by him to be so effectual in India.

2nd. We may use injections of tincture of iodine or solution of ergotin into the substance of the tumour.

3rd. We may divide the isthmus of the thyroid body.

4th. Or we may excise the gland wholly or in part.

I would lay it down as a general rule that no cutting operation should be performed in this variety of enlarged thyroid unless dyspnœa, or dysphagia or aphonia be present, so as to cause serious distress or even danger to the patient.

Even then it is generally wise to try the effect of external remedies and of injections into the tumour before proceeding further.

*Case of simple hypertrophy of the thyroid body in a young lady, causing severe dyspnœa, cured by repeated injections of tincture of iodine into the tumour.*

A. S., aged 21, a dressmaker, came under my care in December, 1885. She had been suffering from an enlargement of the thyroid for several years, but difficulty of breathing had only come on a few weeks before I saw her.

The tumour was of considerable size, involving both lobes, but the right was double as large as the left. Inspiration was noisy and difficult, and if she took any exercise the dyspnœa became alarming. Upon one occasion she was carried into a shop in a very serious condition, and a surgeon who was called in was very nearly performing tracheotomy.

I injected 10 minims of tincture of iodine into the tumour every week for three months, and it steadily diminished in size, while the dyspnœa at the same time grew less. I then lost sight of her for a time, and on seeing her again about a year afterwards I found that the thyroid body was of normal size and the breathing quite easy.

I have treated a large number of cases of simple hypertrophy of the thyroid body by means of injections of iodine and of solution of ergotin, and I am bound to say that in the large majority of cases the result has been disappointing.

In a few, the tumour has diminished, but in the majority there has been no perceptible improvement, even after a long and steady perseverance with the treatment.

It is quite possible that we may yet find a drug that will prove efficacious when injected into the tumour, but at present we are often driven to perform some cutting operation in those cases which are causing severe dyspnœa, or are otherwise endangering life.

I was very much struck with a series of cases reported by Mr. Sydney Jones in the *Lancet* for Nov. 24, 1883, where he cut down upon the isthmus of the enlarged thyroid, and having freed it from the trachea, ligatured it on each side and divided it between; the effect being not only to cure the dyspnœa but also to cause subsequent atrophy of the lateral lobes. I accordingly tried this mode of treatment in the following case, and was much gratified by the result. It was a very bad case, and the result was a perfect cure, brought about by a very simple operation.

*Case of simple hypertrophy of thyroid body in a woman, causing severe dyspnœa, treated by division of the isthmus; cure.*

M. W., aged 54, was sent in to the Bristol Royal Infirmary by Mr. Forty, of Wotton-under-Edge, on Oct.

16th, 1886. She had noticed an enlargement of the thyroid body for about three months, and during the whole of that time there had been dyspnœa, and latterly dysphagia also.

Both lobes were involved, and the isthmus was very large and hard, pressing firmly on the trachea.

Breathing was loud and difficult, especially on the slightest exertion. She could swallow liquids fairly, but solids passed with difficulty. The patient was very thin, but was otherwise in good health.

I tried two or three injections of tincture of iodine, but without any improvement; and, as the patient was anxious for something to be done to enable her to move about, I cut down on the isthmus on October 22nd, and having passed a catgut ligature round each extremity, I tied it tightly at both ends and divided it between, pushing it off from the trachea, which it was firmly compressing.

The result was a surprise to me, for not only did the operation cure the dyspnœa and dysphagia, but the lateral lobes at once began to diminish in size, and when she left the Infirmary on November 3rd there was very little enlargement of the neck visible.

In the two following cases it appeared to me advisable to remove a portion of the tumour rather than merely to divide the isthmus, for in the first case the right lobe was clearly the part which was pressing on the trachea and causing the dyspnœa; and in the other case the isthmus itself was converted into a large median lobe, which it was not safe to divide.

*Case of simple hypertrophy of the thyroid body in a young man, causing severe dyspnœa, treated by excision of one lobe; cure.*

J. S., aged 18, was sent to me at the Bristol Royal Infirmary by Dr. Parry on April 11th, 1888. He was a pupil teacher, and his thyroid body had been slowly enlarging for eighteen months previous to admission. During this time he had been rapidly losing flesh, and for the last few weeks he had been suffering from dyspnœa. For the last three days the difficulty in breathing had been alarming, and he made a loud croupy noise with every inspiration and expiration, which was much increased on taking any exertion.

I made an incision in the median line of the neck on to the thyroid isthmus, and then another at right angles to this over the right lobe, dividing the sterno-hyoid and sterno-thyroid muscles.

The right lobe, being felt to be only loosely attached to surrounding structures, was then shelled out, slight adhesions being broken down by the fingers and the handle of the scalpel. All vessels were first clamped by forceps in two places and then divided between, by which means very little blood was lost. A large pair of forceps was next clamped on the junction of the right lobe and isthmus, preparatory to ligaturing, but on doing this the dyspnœa became alarming, until, the ligature having been tied tightly, the clamp was removed, when the dyspnœa at once ceased. The right lateral lobe was finally cut away with scissors, and the wound brought together with sutures, one small drainage tube being left in.

The patient made an excellent recovery; his difficulty in breathing gradually diminished until two months after the operation, when it entirely disappeared. He gained flesh and improved steadily in every way. When I last saw him, in August, the left lobe was very little larger than the normal size, and the scar over the excised right lobe was scarcely noticeable.

*Case of enlargement of the thyroid body in a young girl, causing severe dyspnœa. Removal of the central portion of the tumour; atrophy of the remainder. Cure.*

E. S., aged 13, was admitted into the Infirmary under my care on October 4th, 1888.

There was a family history of goitre, her maternal grandmother, mother, and one sister all being afflicted with it.

The patient first noticed an enlargement of the thyroid body two years ago, but for the last three or four months it had rapidly increased in size, and during this time she had been gradually suffering more and more from dyspnœa.

Her neck measured fourteen inches in circumference, each lateral lobe of the thyroid body standing out prominently, and in addition there was a considerable swelling in the mid line of the neck and over the trachea, forming a kind of middle lobe. Her dyspnœa was distressing; she could not move across the room without fear of suffocation, and the noise she made during sleep could be heard for some distance.

On October 11th I removed the central portion of the tumour, making a median vertical incision only. I first passed a strong silk ligature around each side of the portion I intended to remove, and then dissected it off from the trachea and the surrounding parts and removed it with very little hæmorrhage. During the operation the breathing was at times very difficult, but it improved as soon as the tumour was completely removed.

She made an excellent recovery. At the present time the wound has healed with the exception of a small sinus at the lower part of the incision where the drainage tube was inserted. The lateral lobes have atrophied to less than half their former size, and they are still diminishing. The breathing is now perfectly tranquil and she can walk and run about without making any of the tracheal noise which so troubled her before. In addition to all these her general condition has much improved since the operation, and she now looks quite healthy and strong, whereas she was before very pale and weakly.

I have never yet had to treat a case of the fourth variety, or fibrous enlargement; but, in these cases nothing is of any avail except excision of the tumour, so I pass on to the fifth variety, or malignant enlargement, a case of which has lately come under my treatment.

A considerable number of cases of primary carcinoma of the thyroid body have now been recorded, but it is comparatively rare, and the following is the only case that ever came under my notice.

*Case of primary carcinoma of the thyroid body in a lady; threatened death from suffocation; excision of the tumour. Death twenty-three days afterwards.*

Mrs. T., aged 45, seen in consultation with Mr. Forty, of Wotton-under-Edge. She had noticed an enlargement of the thyroid for about four months, but it was only during the last two months that any symptoms were felt, these being dyspnœa and loss of flesh. During the last two months the tumour had rapidly increased and had become harder. The dyspnœa had been steadily increasing, and latterly had been extreme. It would come on in paroxysms, and threatened suffocation. Two nights before I saw her it was so bad that her friends thought she would not live through the night, the vessels of the face became congested, and there was œdema of the right side of the head and neck for several hours.

I saw her on October 19, 1888.

There was then a swelling about the size of a duck's-egg over the front and sides of the trachea, but rather more on the right than the left side. It was apparently growing from the isthmus of the thyroid body and was very firmly attached to the trachea. The skin over it was red, somewhat adherent to the parts beneath, and the growth itself was very firmly fixed to the deep parts. There were three or four enlarged glands above the right clavicle and one or two in the right anterior triangle of the neck. Respiration was very difficult, and it was evident that the trachea was much contracted by the tumour. The patient was very thin and weak, and expressed herself as confident that she could not live long unless something could be done to relieve her. I diagnosed it as a case of malignant disease of the thyroid body, and foresaw that removal would be a difficult matter, as the tumour was so firmly fixed to the deep parts.

Tracheotomy was out of the question, as the tumour reached to the upper border of the sternum, and the only possibility of relief to the breathing lay in an attempt to remove the tumour. This I accordingly did, and after considerable difficulty I succeeded in removing the whole of the tumour.

I began by dissecting it up from the trachea below, and as soon as I had made sufficient room I made an incision into the trachea just above the sternum, and inserted a tracheotomy tube; this rendered the breathing, which had been becoming very alarming, at once perfectly easy, and I was enabled to complete the removal in a leisurely manner. The growth was very firmly adherent to the trachea, which was compressed so that only a very small aperture remained; its walls were evidently infiltrated with the growth, which was also invading the neighbouring muscles. I was very ably assisted during the operation by my friend Mr. Forty, and chloroform was very carefully and effectually given by Mr. Simmonds. After the operation I removed the tracheotomy tube and sutured the opening in the trachea. All bleeding vessels having been then tied, I inserted a large drainage tube and closed the skin wound. Shortly afterwards smart hæmorrhage occurred and the breathing again became difficult; so, as I had left to catch a train, Mr. Forty promptly reopened the wound, reinserted the tracheotomy tube, and having tied the bleeding vessel restored her to comfort for the time.

I did not see the patient again, but Mr. Forty informs me that broncho-pneumonia ensued a few days afterwards, and she gradually sank and died on November 11th, twenty-three days after the operation.

Most writers are agreed that malignant enlargements of the thyroid body should be let alone, but in the above case I removed the tumour to save the patient from immediate death by suffocation. If there had been room to perform tracheotomy below the tumour I should have done so, but as this was impossible I could only prolong the patient's life by removing the tumour; and I should not hesitate to operate in a similar manner upon another occasion.

